# Metaplastic Carcinoma of the Breast: Analysis of 38 Cases from a Single Institute

**DOI:** 10.5146/tjpath.2019.01472

**Published:** 2020-01-15

**Authors:** Bermal Hasbay, Filiz Aka Bolat, Hüseyin Özgür Aytaç, Hülya Aslan, Ayşin Purbager

**Affiliations:** Department of Pathology, Başkent University, Dr. Turgut Noyan Application and Research Center, Adana, Turkey; Department of General Surgery, Başkent University, Dr. Turgut Noyan Application and Research Center, Adana, Turkey; Department of Radiology, Başkent University, Dr. Turgut Noyan Application and Research Center, Adana, Turkey

**Keywords:** Metaplastic breast carcinoma, Survival, Immunohistochemistry

## Abstract

***Objective:*** To evaluate the pathological and radiological features, hormone profiles, surgery and treatment methods of metaplastic breast carcinoma cases diagnosed at our center in the light of current literature.

***Material and Method:*** A total of 38 metaplastic breast cancer cases diagnosed between 2006-2018 at our center were included in the study. The patients were evaluated in terms of age, tumor size, localization, histological grade, hormone profiles (ER, PR, Her2-neu), American Joint Committee on Cancer (AJCC) Tumor, Lymph node status, Metastases (TNM) stage, progression, survival, radiological features, types of surgery and therapy modalities (chemotherapy and / or radiotherapy).

***Results:*** The age of the patients ranged between 32 and 95 years. Pathological evaluation of cases showed that 14 were pure epithelial (IC-NST + squamous cell carcinoma) and 24 were metaplastic carcinomas with mesenchymal differentiation. Ductal carcinoma in situ (DCIS) was accompanying an invasive component in twenty cases. Seventeen patients had lymph node metastasis. Twelve patients developed distant metastasis. Thirty patients were triple negative for hormone receptors. The mean follow-up period of the patients was 34 months. The estimated life expectancy was 116 months. All of the patients received chemotherapy and 28 patients received adjuvant radiotherapy. There was no correlation between tumor size and lymph node or distant metastasis in our series. Our findings are consistent with the literature.

***Conclusion:*** Metaplastic breast carcinoma is a rare entity among breast carcinomas. Metaplastic carcinomas of the breast draw attention with the differences in their clinical course and the radiological and pathological heterogeneity.

## INTRODUCTION

Metaplastic breast carcinoma (MBC) is a rare subtype and accounts for 0.2% to 5% of all breast carcinomas (1,2). MBC was first described as a mammary carcinoma with mixed epithelial and sarcomatoid components by Huvos et al. in 1973 (3). The current (2012) World Health Organization (WHO) classification distinguishes five subtypes: low grade adenosquamous carcinoma, fibromatosis-like metaplastic carcinoma, spindle cell carcinoma, squamous cell carcinoma, and metaplastic carcinoma with mesenchymal differentiation (chondroid, osseous and other types of mesenchymal differentiation) (2,4).

MBC is a distinct group of breast cancer, in which adenocarcinoma co-exists with a mixture of spindle cells and squamous, chondroid or bone-forming neoplastic cells, and differs from the classical invasive ductal or lobular carcinoma regarding its incidence, pathogenesis and prognosis (1,5). These non-adenocarcinoma elements may be present as a microscopic foci or may dominate the histologic pattern (5). The molecular mechanism of metaplastic carcinoma differs from other types of breast carcinomas, including basal-like breast carcinomas (5,6).

It is suggested that upregulation of cancer stem cell (CSS) and epithelial-mesenchymal transition (EMT) genes might play a crucial role in the pathogenesis of MBC (1,5,7). EMT activators and CSS present especially in the non-glandular components of metaplastic carcinomas (8).

Due to its rarity, there is limited data correlating the imaging features with clinical presentation and the histopathologic features of MBC (9–11). Metaplastic cancers were previously radiologically defined as benign lesions (9,10). Metaplastic carcinomas radiologically demonstrate benign features compared to invasive ductal carcinomas such as an oval or rounded shape, circumscribed margins and lack of malignant calcification (12).

MBC cases are typically negative for hormone receptors and do not exhibit Her2-neu overexpression (1,13,14). Even though MBC is similar to triple negative breast cancers (TNBC) for receptor status, its molecular features are different and the clinical outcomes are even worse than for TNBC (1,14).

The aim of this study was to evaluate metaplastic carcinoma, a rare subtype of breast tumors, in terms of histopathological features, hormone receptor status, radiological features and treatment modalities.

## MATERIAL and METHODS

A total of 38 MBC patients diagnosed between 2006 and 2018 at Baskent University Faculty of Medicine, Adana Research Hospital were included in the study. Ethics committee approval was received for this study from local ethics committee. A 12-year electronic data search was performed in the laboratory information system using the keywords ‘’metaplastic carcinoma‘’ plus ‘’breast’’ in the diagnostic line. Thirty-eight cases met the criteria based on pathology reports and/or review of slides. Cases showing a metaplastic tumor component were included in the study. The patients were evaluated retrospectively for age, tumor size, tumor localization, histological grade, hormone receptor status (ER, PR, Her2-neu), American Joint Committee on Cancer (AJCC) Tumor, Lymph node status, Metastases (TNM) stage, progression, recurrence, survival, radiological features, surgery, and treatment modalities (adjuvant, neoadjuvant: chemotherapy and / or radiotherapy).

All of the cases were re-reviewed according to the 2012 WHO classification. Clinicopathological and demographic features were evaluated in detail. The pathologic diagnosis of MBC was made by two pathologists who were specialized in breast pathology and the sonographic and MRI features were assessed by two breast radiologists.

Immunohistochemical (IHC) assays were performed using monoclonal antibodies against ER (Clone EP1, Code M3643, Dako, Denmark) and PR (Clone Y85, 60-0056-7, Genemed, Germany) and Her2-neu (Code A0485, Dako, Denmark). ER and PR were prepared by taking positive and negative control tissues and using ready-to-use solutions in the Leica Bond Max device. We followed the ASCO and CAP recommendations for reporting the results of the IHC assays for ER, PR and Her2/neu. For ER and PR, all cases with at least 1% positive cells were considered as receptor positive (5,15). The Allred score, which combines the percentage of positive cells and the intensity of the reaction, was used for ER-PR evaluation (16).

Her2-neu status can be determined by assessing protein expression on the membrane of tumor cells using IHC or by assessing the number of Her2-neu gene copies using in situ hybridization (ISH). The results for Her2-neu testing by IHC were reported according to the intensity and the percentage of positive staining in tumor cells (0, 1+, 2+, 3+). Scores of 0 and 1+ were considered as negative for Her 2-neu amplification. A score of 3+ was considered as positive. A score of 2 was considered as equivocal and ISH was ordered for confirmation. Her 2 was considered to be amplified if the average Her2-neu copy number was ≥6 signals/cells or the Her2/CEP 17 ratio was ≥ 2 (5,17).

Statistical analysis was performed using the SPSS statistical package software (Version 17.0, SPSS Inc., Chicago, IL, USA). For each continuous variable, normality was checked by Shapiro-Wilk tests and by histograms. All numerical data were expressed as median values (Minimum-Maximum) or as proportions. Comparisons between groups were evaluated using the Mann-Whitney U test and the Kruskal-Wallis test was used for the data not normally distributed.

The association with overall survival was analyzed using the Wald test and the log-rank test was used to examine their relationship when different variables were applied. The survival curve was plotted using standard Kaplan-Meier methodology.

Written consent was not obtained from the patients since the study was designed retrospectively and needed no consent.

## RESULTS

The age of the patients ranged from 32 to 95 years and the mean age was 55.34 ± 14.08 years. The left breast was involved in 22 of the 38 (57.9%) patients and the right breast in 16 (42.1%). The mean tumor diameter was 4.48 ± 2.53 cm (max. 11.5, min. 1.8 cm). Of the 38 cases, 7 were dead, 31 were alive. The mean age of the surviving patients was 53.74 ± 12.98 years (32-95) and the mean tumor size was 4.46 (1.8-11.5) cm.

**Figure 1:**
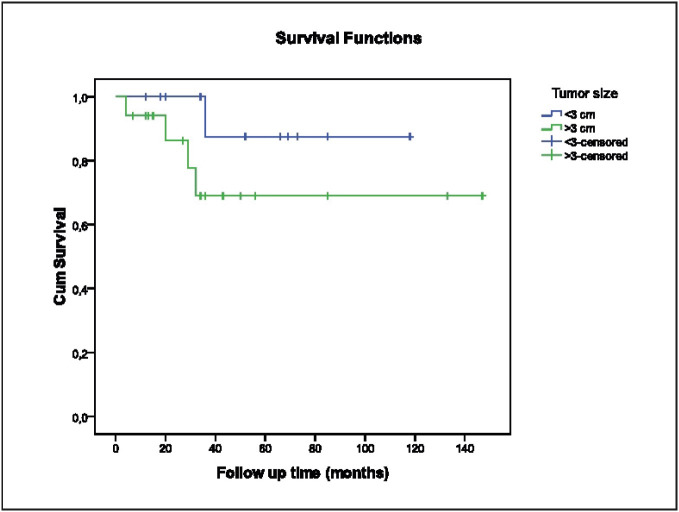
Relationship between tumor size and survival (p= 0.217).

The estimated life expectancy of all patients was 116.3 ± 10.2 months (95% CI 96.3-136.3); 1-year survival 94.7%; 3-year survival 75%; 5-year survival 75%. While the estimated mean life expectancy of those with a tumor size ≤ 3 cm was 107.7 ± 9.6 months (95% CI 88.9-126.5), for those with a tumor size > 3 this was found to be 108.5 ± 11.1 months (95% CI 76.7-140.3) (p= 0.217 log-rang test) (Figure 1). Therefore, tumor size below 3 cm or above 3 cm had no effect on survival.

The mean age of the dead patients was 62.43 ± 17.57 years (46-95) and their mean tumor size was 4 (3-5) cm. Tumor histological grade was three for all patients. Eighteen of our patients underwent a mastectomy, of which 15 had axillary dissection and three had sentinel lymph node biopsy (SLNB). While one of the other 17 patients underwent only segmental mastectomy, 16 patients had segmental mastectomy and SLNB. A patient who was diagnosed with metastatic axillary lymph node at the age of 95 had only axillary lymph node sampling. Two patients were diagnosed from paraffin blocks as consultation cases and then were out of follow-up.

In the radiological examination of the patients, twenty-two patients underwent ultrasound where seventeen had malignant appearing solid masses, one had a mass of suspected malignancy, three had well-defined solid masses and one had an appearance compatible with mastitis. Eighteen patients had mammograms and MRI, where 12 showed solid masses of malignant appearance with irregular margins, asymmetric opacity and intermittent microcalcifications. In three cases with well-limited nodules on ultrasound, MRI showed solid lesions suspicious for malignancy.

**Figure 2:**
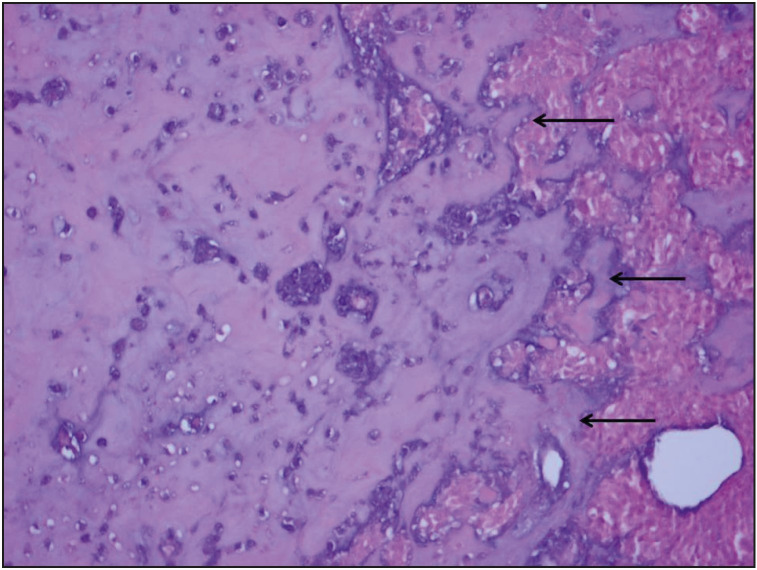
Epithelial component and chondroosseous areas (arrows) (H&E; x200).

**Figure 3:**
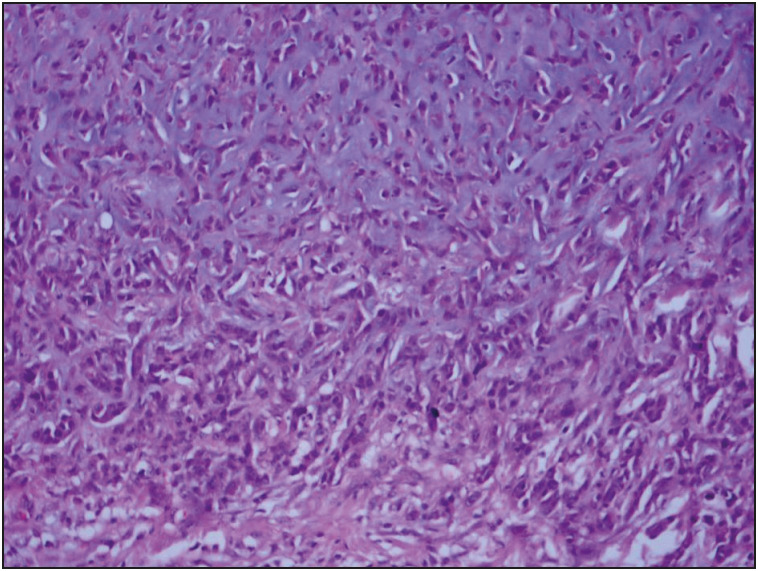
Matrix producing type carcinoma areas (H&E; x200).

**Figure 4:**
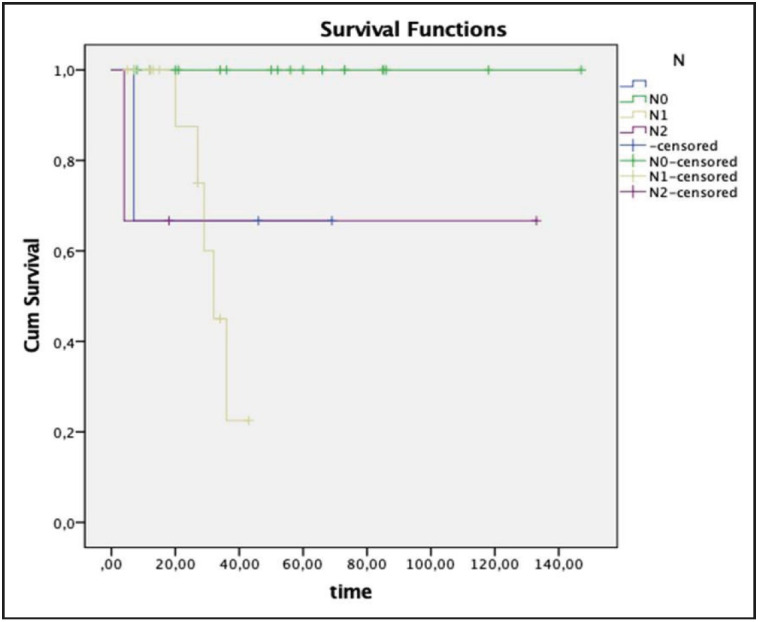
Relationship between pathological node (pN) values and survival (p= 0.009).

Pathological results of the cases were as follows: pure epithelial carcinoma (IC-NST + squamous cell carcinoma) in 14 cases, mesenchymal component metaplastic carcinoma in 24 cases: (carcinoma including chondroosseous areas (Figure 2), mixed carcinoma (two pleomorphic sarcoma, one chondrosarcoma, one leiomyosarcoma), matrix-producing type carcinoma (Figure 3) and carcinomas with squamous and spindle cell areas cases). Ductal carcinoma in situ was accompanying an invasive component in twenty cases. Of these, the most common was the solid type, followed by the comedo and cribriform types. Axillary lymph nodes were observed to be benign in 18 of 35 patients (51%), and metastatic in 17 of 35 (49%). Among those with metastatic axillary nodes, 14 cases had pN1 and 3 cases had pN2. In terms of pN, 1-year survival was 100% for pN0, 87.5% for pN1, 66% for pN2 with a significant p value (0.009) (Figure 4). Twelve patients developed distant metastasis. Four patients had lung metastasis, two had supraclavicular lymph node metastasis, one had mediastinal lymph node metastasis, one had bone, liver and lung metastasis, two had bone metastasis and one had brain metastasis. Mean life expectancy of M0 (without metastasis) patients was 128.6 ± 9.9 months (95% CI 109.1-148.0) while the figure for M1 (with metastasis) patients was 55.7 ± 10.8 month (95% CI 34.6-76.9) (p = 0.077 log-rang test). In our series of 38 cases, two patients had ER positive, one had ER and PR positive, one had ER and Her2-neu positive, four had only Her2-neu positive tumors and the remaining 30 had tumors negative for all receptors. A summary of the hormone receptor profiles of the cases can be seen in Table 1.

**Table 1:** Summary of hormone receptor positive cases.

**Cases**	**Diagnosis**	**ER**	**PR**	**Her 2-neu**
1	IC-NST + SCC	30-40% Positive	Negative	Negative
2	IC-NST + SCC	40-50% Positive	Negative	Negative
3	Containing squamous and spindle cell areas	30-40% Positive	Negative	Score of 3
4	IC-NST + SCC	60-70% Positive	15-20% Positive	Negative
5	IC-NST + SCC + containing spindle cell areas	Negative	Negative	Score of 3
6	IC-NST + SCC	Negative	Negative	Score of 3
7	IC-NST + SCC	Negative	Negative	Score of 3
8	IC-NST + SCC	Negative	Negative	Score of 3

**IC-NST:** Invasive carcinoma-Carcinoma of no special type, **SCC:** Squamous cell carcinoma, **ER:** Estrogen receptor, **PR:** Progesterone receptor.

Mean follow-up period of patients was 34 months and ranged between 4 and 147 months. Five of the patients who died had pT2 and two had pTx tumors. All the patients who had pT1c, pT3 and pT4 survived. A detailed summary of dead patients is provided in Table 2.

**Table 2:** The features of the 7 dead patients.

	**Age**	**Tumor size**	**Lymph node stage**	**Metastasis**	**Surgical procedure**	**Hormone profile**	**Follow-up time (months)**	**Treatment**
1	48	3.5 cm	N1	Lung	M + ALND	Negative	20	CT + RT
2	46	4 cm	N1	M0	BCS + ALND	Negative	29	CT + RT
3	59	Ready block	-	Lung	-	Negative	7	CT + RT
4	95	-	N1	M0	ALND	Negative	27	-
5	54	4.5 cm	N2	M0	M + ALND	Negative	4	CT
6	58	5	N1	Bone + lung + liver	BCS	Negative	32	CT + RT
7	77	3	N1	Bone	M	Negative	36	CT

**M:** Mastectomy, **BCS:** Breast conserving surgery, **ALND:** Axillary lymph node dissection, **SLND:** Sentinel lymph node dissection, **CT:** Chemotherapy, **RT:** Radiotherapy.

Among the patients that we followed-up, seven had chemotherapy (CT); three had chemotherapy, radiotherapy (RT) and Transtuzumab; two had CT, RT and Tamoxifen; and 24 had CT and RT. As a result, all patients were treated with CT, and 28 of them also received RT. When viewed in terms of treatment, there was no difference in survival between CT or CT+RT and the p value was 0.391. The clinico-pathological characteristics of the 38 patients with MBC are detailed in Table 3.

**Table 3:** Clinico-pathological characteristics of 38 patients with MBC. [n (%)]

**Age group** ≤ 50 14 (36.8) > 50 24 (63.2) Mean age 55.34 (32-95)	**Her2-neu** Positive 5 (13.2) Negative 33 (86.8)
**Tumor location** Right 16 (42.1) Left 22 (57.9)	**pN** pN0 18 (47.4) pN1 14 (36.8) pN2 3 (7.9) pN3 0 (0) pNx 3 (7.9)
**Tumor size** ≤ 3 cm 13 (34.2) >3 cm 17 (44.7) Unknown 8 (21.1) Mean size 4.48 cm (1.8-11.5 cm)	**Tumor subtype** IC-NST + SCC 14 (36.8) Mixed carcinoma 8 (21.1) Pleomorphic sarcoma Chondrosarcoma Leiomyosarcoma Squamous and spindle cell areas Matrix producing 4 (10.5) MMC 12 (31.6)
**pT** pT1 3 (7.9) pT2 20 (52.6) pT3 4 (10.5) pT4 3 (7.9) Unknown 8 (21.1)	**Metastasis** Lung 4 (10.5) Lymph node 3 (7.9) Bone+liver+lung 1 (2.6) Bone 2 (5.3) Brain 1 (2.6) No metastasis 27 (71.1)
**Nuclear grade** G1 0 G2 0 G3 38 (100)	**Surgery** Mastectomy 18 (47.4) and ALND 15 and SLND 3 Segmental mastectomy 17 (44.8) and SLND 16 ALND 1 (2.6) Unknown 2 (5.2)
**Estrogen receptor (ER)** Positive 4 (10.5) Negative 34 (89.5)	**Systemic therapy** CT only 7 (18.4) CT + RT 24 (63.1) CT + RT + TTZ 3 (7.9) CT + RT+ TMX 2 (5.3) Not followed 2 (5.3)
**Progesterone receptor (PR)** Positive 1 (2.6) Negative 37 (97.4)	**Final status** Alive 31 (81.5) Ex 7 (18.5)

**SLND:** Sentinel lymph node dissection, **CT:** Chemotherapy, **RT:** Radiotherapy, **MMC:** Mesenchymal component metaplastic carcinoma, **ALND:** Axillary lenf node dissection, **pT:** Pathologic tumor stage, **pN:** Pathologic nodal stage, **IC-NST:** Invasive carcinoma-Carcinoma of no special type, **TTZ:** Transtuzumab, **TMX:** Tamoxifen.

## DISCUSSION

Metaplastic breast carcinoma has a poor prognosis and is usually triple negative. MBC pathologically comprises different histologic components of both epithelial and mesenchymal origins (1,2). It constitutes between 0.2% and 5% of all breast cancers and is generally observed in the sixth decade of life (2). The mean age of our patients was 54 years, which was consistent with the literature. Patients with MBC usually present with large size, higher grade and hormone receptor negative tumors (13,14). Similarly, mean tumor size of our cases was 4.48 (1.8-11.5) cm, all of them were histologically grade 3, and 79% had a negative hormone profile. Three of the cases were pT1c, 20 were pT2, four were pT3, and three were pT4. Another 7 patients were evaluated as pTx because three of them were sampled after chemotherapy, and four were diagnosed using preformed archival paraffin blocks of different centers. Even though MBC is usually reported to be an aggressive tumor with fewer nodal metastases (1,14), there are some studies demonstrating a frequency of nodal metastases of up to 21% to 64% (1,18,19). In our series, 17 patients (49%) had axillary lymph node metastasis which was consistent with the literature. Thirteen of seventeen patients with lymph node metastasis had pure epithelial component, one patient had carcinosarcoma and three patients had metaplastic carcinoma with mesenchymal differentiation.

Ductal carcinoma in situ may be visible adjacent to metaplastic carcinoma at a rate of 11% to 65%. The presence of DCIS strongly supports the diagnosis of metaplastic carcinoma (20). In our series, 20 of 38 (52.14%) cases had DCIS which was also consistent with the literature.

MBC is a heterogeneous disease with different subgroups. According to the 2012 WHO classification, it is divided into 5 groups: low grade adenosquamous carcinoma, fibromatosis–like metaplastic carcinoma, squamous cell carcinoma, metaplastic carcinoma with mesenchymal differentiation (chondroid, osseous, other types of mesenchymal differentiation) and spindle cell carcinoma (2,4). We did not observe any low grade adenosquamous carcinoma or fibromatosis-like metaplastic carcinoma in our series.

Several hypotheses have been suggested for the etiopathogenesis of MBC. The first one is the ‘cancer stem cell hypothesis’, which describes the cells that have the capacity to self-renew and differentiate into different cell types (21,22). The carcinomatous and sarcomatous components may develop from separate progenitor cells or both components may develop from multipotential progenitor cells. One other theory is related to the changes in the expression of membrane proteins involved in cell polarity and in the tight linkage functions between cells which is called ¨Claudin¨ (22).

Optimal treatment of MBC is in the same way as IC-NST. Surgery is the main curative approach. Mastectomy or breast conserving surgery have been the most commonly performed procedures (23,24). All of the patients received surgical treatment in our series. The most common surgical procedure was the modified radical mastectomy, which was performed in 18 patients. Seventeen patients had breast conserving surgery. All of our patients were treated with chemotherapy after surgery and 28 of them received additional radiotherapy.

Hormonal therapy generally has no role in the management of patients with MBC. There is a high incidence of hormone receptor negativity as well as lower Her2-neu overexpression in MBC. In our series of 38 patients, only four were hormone positive and five were Her2-neu positive, and the rest of them were negative hormone receptors. Her2-neu overexpression rate has been variable in the literature, between 2% and 26% (13,14,20,25). ER positivity varies between 6% and 12% in various studies (20). In our series, only 5 of 38 (13%) cases had positive Her2-neu and 4 of 38 (10.52%) cases had positive ER which is also consistent with the literature. In the study of Rakha et al., these three markers were more often positive in squamous carcinomas (13). In our cases, all ER and Her2-neu positive cases were metaplastic carcinomas with squamous cell carcinoma (Table 1). Tamoxifen was added to the treatment of ER positive patients, and Transtuzumab for Her2-neu positive patients.

The median follow-up for MBC patients was 34 (range 4-147) months. Among patients who developed distant metastasis during follow-up, the lung was the most common site as seen in four patients. Bone metastasis was seen in two patients, supraclavicular lymph node involvement in two patients, mediastinal lymph node metastasis in one patient, both bone, liver and lung metastasis in one patient and brain metastasis in one patient.

Diagnosis of MBC cannot rely on imaging features alone. Core needle biopsy and aspiration cytology may aid in the pre-operative diagnosis, but the probability of misdiagnosis would increase in the presence of hemorrhage or necrosis due to inadequate sampling or a poor choice of puncture region (12,26). Excisional biopsy is the gold standard without doubt and should be used in all patients prior to surgery.

If core biopsy shows the appearance of a pure malignant mesenchymal tumor, this may be a primary malignant mesenchymal tumor of the breast, a malignant phyllodes tumor or a metaplastic carcinoma with mesenchymal differentiation. Once the hematoxylin and eosin stained slides are examined, a decisioni can be made as follows: If the tumor was pure sarcomatoid, then it had to express at least 2 epithelial markers in the immunohistochemistry analysis (cytokeratin 5/6, high molecular weight cytokeratin, P63, pancytokeratin, CK7) for the diagnosis of MBC. According to the histopathological appearance of the sarcomatoid component, it is possible to add S-100, Smooth Muscle Actin (SMA), CD68, Calponin, and Desmin. It is more appropriate to provide a definite diagnosis on excisional biopsy specimens. In the majority of cases, the transition foci between MBC and IC-NST were only observed following surgical biopsies (12,26). Metaplastic carcinoma should also be considered when a spindle cell lesion is seen on a breast tru-cut biopsy. Extensive sampling from the surgical biopsy and an immunohistochemical examination should therefore be performed to avoid misdiagnosis.

The differential diagnosis between MBC, sarcoma and phylloides tumor is important as SLNB sampling is mandatory for MBC, but not for others.

In conclusion, MBC is a rare entity among breast carcinomas. It is an aggressive tumor that is more likely to present with worse prognostic indicators such as tumor size and stage. However, low-grade adenosquamous carcinoma in this group has a good prognosis.

The diagnosis of MBC is difficult in some cases and requires rigorous use of immunohistochemistry. Most of the cases present with poor prognostic indicators and show lack of expression of hormone receptors as well as Her2-neu. It is evident that more studies are needed to understand the true biologic potential of this tumor compared with other forms of breast carcinoma. In conclusion, our findings are consistent with the literature. Prospective multi-center wide scale studies should be carried out in the future to cast light on the clinical and pathologic aspects of MBC.

## Conflict of Interest

No conflict of interest was declared by the authors.

## FUNDING

The authors declared that this study has received no financial support.
